# Reducing Anemia Among School-Aged Children in China by Eliminating the Geographic Disparity and Ameliorating Stunting: Evidence From a National Survey

**DOI:** 10.3389/fped.2020.00193

**Published:** 2020-05-12

**Authors:** Jun-Yi Wang, Pei-Jin Hu, Dong-Mei Luo, Bin Dong, Yinghua Ma, Jie Dai, Yi Song, Jun Ma, Patrick W. C. Lau

**Affiliations:** ^1^Institute of Child and Adolescent Health, Peking University School of Public Health, National Health Commission Key Laboratory of Reproductive Health, Beijing, China; ^2^Department of Sport and Physical Education, Hong Kong Baptist University, Kowloon Tong, China

**Keywords:** anemia, geographic disparity, stunting, children, China

## Abstract

**Background:** The aim of this study was to assess the geographic disparity in anemia and whether stunting was associated with anemia in different geographic groups among school-aged children in China.

**Methods:** 71,129 Han children aged 7, 9, 12, and 14 years old were extracted from the 2014 cycle of Chinese National Surveys on Children Constitution and Health. Anemia, anemia severity, and stunting were defined according to WHO definitions. Binary logistic regression models were used to estimate the association between anemia and stunting in different geographic groups.

**Results:** The prevalence of anemia was significantly higher in girls (10.8%) than boys (7.0%). The highest anemia prevalence was in Group VII (lower class/rural, 12.0%). A moderate/severe prevalence of anemia was concentrated in Group VII and Group VIII (western/lower class/rural) for both sexes. The prevalence of anemia was higher in stunting boys than non-stunting boys in Group IV (lower class/city, χ^2^ = 12.78, *P* = 0.002) and Group VII (χ^2^ = 6.21, *P* = 0.018), while for girls, it was higher in stunting girls than their non-stunting peers only in Group II (upper class/large city, χ^2^ = 4.57, *P* = 0.046). Logistic regression showed that the stunting children have 30% higher risk of anemia than non-stunting children after adjustment for age, sex and school (OR = 1.30, 95% CI: 1.05–1.60).

**Conclusion:** A significant geographic disparity and an association between anemia and stunting among specific groups of school-aged children in China was demonstrated. Consequently, eliminating the geographic disparity and ameliorating stunting might contribute to the improvement of Chinese children's anemia. Specific guidelines and interventions are needed, especially for adolescent girls and the groups with serious anemia burden.

## Introduction

Anemia, or low concentrations of hemoglobin (Hb), adversely affects cognitive and motor development and study capacity, and increases susceptibility to infection, which also exerts a substantial economic burden on the government (1, 2). Globally, 1.62 billion people suffer with anemia and the prevalence among children was 25.4%, according to a World Health Organization (WHO) (3) report. Asia is the hardest hit, especially in South Asia (4, 5), where an improvement in children's hemoglobin status may lead to a modest global increase in mean hemoglobin and a reduction in anemia prevalence. Globally, anemia is affecting people in both developed and developing countries with different health risks, and almost all age groups and both sexes are susceptible. Even for developed countries, such as Sweden, France, Australia, Denmark, Belgium, and Ireland, anemia prevalence has changed little during past two decades (6). Although iron deficiency anemia is the true indicator of poor nutritional status, considering that 90–95% of anemia cases in China are due to iron deficiency (7), anemia remains useful as an indicator of undernutrition and is particularly relevant for adolescents in the context of rapid growth and menstruation. Compared with children under five and pregnant women, adolescents have not been paid much attention. In 2016, 333 million adolescents with anemia lived in multi-burden settings, of which 194 million lived in India and China according to GBD (Global Burden of Disease study) data (6). Although the prevalence of anemia in China has declined in the past two decades at the national level (8), it was as high as 14.8% in some rural regions like Shanxi (9, 10). Nationwide, more than 19.2 million Chinese children were anemic and 389,198 had severe anemia in 2010, based on a total number of children aged 7–18 years (194,599,052). Nevertheless, updated information of geographic disparity on anemia among Chinese children is unclear. This information is urgently needed as it may provide a solid foundation to alleviate the geographic disparity of anemia among Chinese children.

In China, most anemia is due to prolonged iron deficiency, which impairs hemoglobin production and limits the amount of oxygen that red blood cells carry throughout the body and to the brain (11). Stunting is also a big issue in China and warrants monitoring because it is an undervalued indicator which reflects the cumulative effect of undernutrition with socioeconomic and other factors. These factors may contribute to anemia. Studies in Haiti and Angola confirmed that stunting increased the risks of developing anemia (12, 13). The question is still uncertain as to whether stunting is associated with anemia in China, especially in school-aged children who have been largely under-investigated. Moreover, if the association exists, does it vary or remain similar in different settings and populations?

Hemoglobin data from children aged 7 to 14 of both sexes across China was collected in the 2014 Chinese National Surveys on Students' Constitution and Health (CNSSCH). It provided a valuable opportunity to update the available information on anemia among school-aged children who have been largely neglected in research in China, compared with numerous publications on children under five and pregnant women (14). The objectives of the present study were to: (1) delineate the geographic disparity of anemia; (2) identify the most susceptible population with the heaviest burden of anemia; and (3) examine the association between anemia and stunting, and the relationships across different geographic groups.

## Materials and Methods

Data was extracted from the 2014 cycle of CNSSCH, which was a large-scale cross-sectional survey of school-aged children conducted by six relevant ministries including the Ministries of Education, Health, Science and Technology, the State Ethnic Affairs Commission, and the State Sports General Administration, China. It spanned 31 provinces, excluding Hong Kong, Macau, and Taiwan. The sampling procedure, as previously described in detail (14), was the same in all CNSSCH survey sites. In brief, this survey was to investigate children's health status in China and used a multistage stratified cluster sampling design (14). In the first stage of sampling, in order to achieve better representation within the 30 provinces, populations were stratified by three socioeconomic indicator groups or three sets of prefecture-level cities (i.e., upper, moderate, low) at the regional level, defined by regional gross domestic product, total yearly income per capita, average food consumption per capita, natural growth rate of the population, and the regional social welfare index. In each group of three sub-provincial levels, one city was selected randomly and remained constant from the first survey in 1985. Within these sub-province regions, populations were also stratified by urban and rural area of residence. Within these stratified areas, a random selection of schools, including primary school, middle school, and high school, was conducted according to the established procedures. In the second stage, sampling took place in classes (primary sampling units or clusters) selected randomly from each grade in these schools, and all students in the selected class were included and listed in the investigation after meeting the inclusion criteria and after obtaining verbally informed consent from both students and their parents. Finally, within the primary sampling units, namely every age from 7 to 18 years for boys and girls, at least 50 Han ethnicity students, the minimum sample size, were included in the survey and sampling yielded equal numbers of the three socioeconomic indicator groups. Thus, the sample weight remained consistent in each age, sex, region (urban/rural), city (three socioeconomic indicators' groups at sub-province level), and province for students aged 7–18 years in each survey year. The participants in this study were Han children aged 7, 9, 12, and 14 years old from 26 provinces and four municipalities, except for Tibet (where the Han ethnicity is a minority). The children were recruited in this study if their parents and themselves had lived in their local regions more than a year.

Participants underwent a medical examination prior to the national survey and were excluded if they had one or more of the following conditions: (1) serious organ disease (e.g., heart, lung, liver, kidney); (2) abnormal physical development (e.g., pygmyism, gigantism); (3) physical impairment or deformity (e.g., severe scoliosis, pectus carinatum, limp, genu valgum, and gunu varum); or (4) acute disease symptoms (e.g., diarrhea, fever) during the past month and not yet recovered. Consequently, 71,129 children with complete data records on age, sex, urban/rural groups, height, weight and hemoglobin concentration were included in the analysis. Moreover, the ratio of boys/girls or urban/rural groups was approximately equal to 1:1 of each sex- and age-specific subgroup. The project was approved by the Medical Research Ethics Committee of Peking University Health Science Center (IRB00001052-18002). With data collected from schools across China, the school principals were able to determine the process for gaining informed consent from children's parents or guardians. All participants' information was anonymized and de-identified prior to analysis to protect their privacy.

### Measures

Participants were required to wear light clothing and stand straight, barefoot, and at ease when height was measured. The heel, humerus, and shoulders were contacted to form a “three-point, one-line” standing position. Measurements were conducted by a team of trained field professionals who were required to pass a training course in anthropometric measurements. Height was measured to the nearest 0.1 cm with a portable stadiometer. The stadiometers were calibrated before use. The measurements were carried out at the same time of the day during the survey (better to be specific, e.g., morning, afternoon, during school recess, etc.). Height-for-age Z-score (HAZ) was calculated by using WHO 2007 references with the fixed population. Stunting was defined using the growth references of HAZ: stunting: < −2SD (15, 16).

Hemoglobin concentration was measured by laboratory technicians for the participants in the selected school. Hemoglobin concentration was measured by HemoCue201+ (Origin: Sweden, Model: HemoCue201^+^, Manufacturer: HemoCue AB) (17). Data collection was supervised as follows by well-trained on-site investigators: (a) the capillary blood sample was collected from the fingertip after discarding the first drop, and a small amount of blood was pressed out onto the fingertips. The blood was taken continuously with a micro cuvette, (b) the micro cuvette was inserted into the cuvette tank and reacted for 15s to 60s, (c) the score was determined and recorded on site (18). Age specific cut-off values of hemoglobin concentration were used to define anemia. Hemoglobin concentration was defined by using WHO criteria (19, 20) and categorized as: (1) for children aged 5 to 11 year: ≥115 g/L normal, <115 g/L anemia, 110–114 g/L mild anemia, ≤ 109 moderate/severe prevalence anemia; (2) for children aged 12–14 year: ≥120 g/L normal, <120 g/L anemia, 110–119 g/L mild anemia, ≤ 109 g/L moderate/severe prevalence anemia.

**Figure 1:**
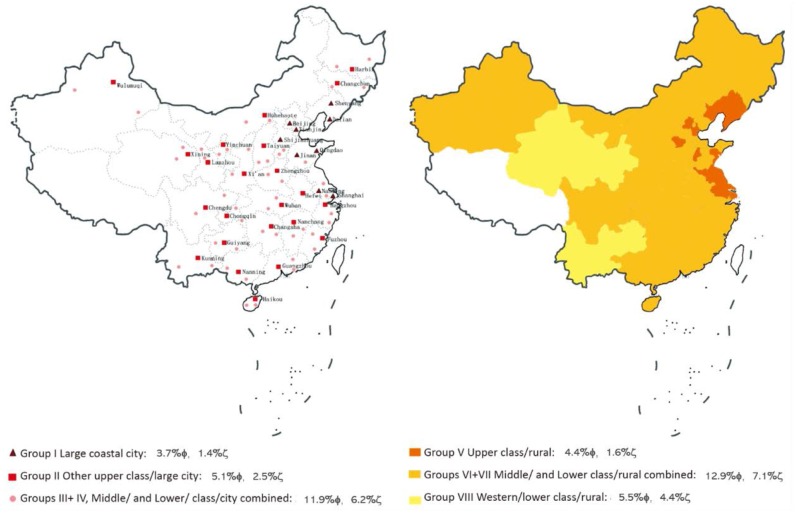
The distribution of different geographic groups with different socioeconomic status (SES); note: ϕ the prevalence of mild anemia in 2014, ζ the prevalence of moderate/severe anemia in 2014.

In order to compare the prevalence of anemia in different geographic groups with different socioeconomic status (SES), all subgroups were further divided into eight categories (21): Group I (large coastal city), Group II (upper class/large city), Group III (middle class/city), Group IV (lower class/city), Group V (upper class/rural), Group VI (middle class/rural), Group VII (lower class/rural), and Group VIII (western/lower class/rural). Group I included the nine largest cities (Beijing, Shanghai, Tianjin, Shijiazhuang, Shenyang, Dalian, Jinan, Qingdao, and Nanjing) and Group II represented the upper urban class. Group VIII constituted the other extreme: rural regions in western provinces, home to the lowest SES class (data was shown in Figure 1).

### Statistical Analyses

The prevalence of anemia in the Han children was described by sex, age, and geographic groups. Chi-square test was used to assess the difference of the prevalence and categories of anemia among different subgroups, while the inspection level was adjusted by Bonferroni method [α′=2α/k(k−1), k = 7]. To assess the geographic difference of association between anemia and stunting, binary logistic regression analyses were conducted with adjustments for sex and age. The design effect of cluster sampling by school was also added in the model. All analyses were conducted using Stata 12.1 (Stata Corp, College Station, Texas). A 2-sided *P*-value of <0.05 was considered significant.

## Results

### The Prevalence of Anemia in Different Geographic Groups

As shown in Table S1, the prevalence of anemia was significantly higher in girls (10.8%) than boys (7.0%). The highest anemia prevalence was in Group VII (12.0%). The lowest prevalence of anemia was observed in Group I (5.2%), Group V (6.0%), and Group II (7.6%). The prevalence of anemia in Group I was more than twice that of Group VII. Table S2 demonstrates that the moderate/severe prevalence of anemia was concentrated in Group VII and Group VIII for both sexes. The prevalence of anemia at each age group was similar to the total sample and the highest prevalence of anemia was found in the age 7 group, followed by 12 years, and the lowest prevalence was the 14-year-old boys group. For girls, those aged 12 and 14 years had significantly higher anemia prevalence than those of 7 and 9 years (data was shown in Table S3).

### The Prevalence of Anemia in Susceptible Population

Girls had a higher prevalence of anemia than boys across different groups. The prevalence of anemia among girls in Group VII was 4–5 times higher than boys in Group I (Table S1). Table 1 and Table S4 indicate that the prevalence of anemia among stunting children was higher than their non-stunting counterparts. When stratified by sex, the prevalence of anemia was higher in stunting boys than non-stunting boys in Group IV (χ^2^ = 12.78, *P* = 0.002) and Group VII (ψ^2^ = 6.21, *P* = 0.018), while for girls, the anemia prevalence was higher in stunting girls than their non-stunting peers only in Group II (χ^2^ = 4.57, *P* = 0.046).

**Table 1:** The prevalence of anemia stratified by stunting status, geographic group, and sex among Chinese school-aged children.

	**Boys**						**Girls**					
**Group^**^#^**^**	**Non-stunting**		**Stunting**		**χ^**2**^**	***P***	**Non-stunting**		**Stunting**		**χ^**2**^**	***P***
	***N***	**%**	***N***	**%**			***N***	**%**	***N***	**%**		
I	98	3.3	1	5.0	13.39	0.066	207	6.9	0	0.0	0.67	1.000
II	299	6.6	4	14.8	2.92	0.100	388	8.5	6	19.4	4.57	0.046
III	374	7.6	8	13.8	3.06	0.084	619	12.7	13	18.6	2.10	0.150
IV	355	6.8	12	17.9	12.78	0.002	454	8.6	10	14.3	2.80	0.129
V	118	4.0	0	0.0	0.29	1.000	240	8.0	1	11.1	0.11	0.531
VI	295	6.7	8	8.6	0.51	0.408	397	9.1	15	15.0	4.04	0.053
VII	710	9.0	15	6.9	1.05	0.395	1201	15.2	29	12.0	1.92	0.202
VIII	161	8.1	22	13.8	6.21	0.018	225	11.3	19	11.6	0.01	0.898
Total	2,410	6.9	70	11.1	16.89	<0.001	3731	10.7	93	13.4	5.11	<0.001

# *Group I (large coastal city), Group II (upper class/large city), Group III (middle class/city), Group IV (lower class/city), Group V (upper class/rural), Group VI (middle class/rural), Group VII (lower class/rural), and Group VIII (western/lower class/rural). Group I included the nine largest cities (Beijing, Shanghai, Tianjin, Shijiazhuang, Shenyang, Dalian, Jinan, Qingdao and Nanjing) and Group II, represented the upper urban class. Group VIII constituted the other extreme: rural regions in western provinces, home to the lowest SES class*.

### The Association Between Anemia and Stunting Stratified by Geographic Groups and Sex

As shown in Table 2, the stunting children have a 30% higher risk of anemia than non-stunting children (OR = 1.30, 95% CI: 1.05–1.60). When stratified by geographic group, the association strengths ranged from 1.41 to 2.34 in Group II, III, IV, VI, and VIII but with 95% CI overlap, while there was no significant association between anemia and stunting in Group I, V, and VII. After stratifying by sex, stunting boys were 1.74 times more likely to be anemic than non-stunting boys while there was no significant association between stunting and anemia among girls. The results of the associations by age stratification were consistent with total sample (data not shown).

**Table 2:** Association between anemia and stunting stratified by geographic group and sex among Chinese school-aged children.

**Group^#^**	**Total^a^**	**Boys^b^**	**Girls^b^**
I	1.17 (0.11–12.63)	29.20 (1.81–471.90)^*^	-
II	2.32 (1.24–4.36)^*^	2.78 (1.15–6.71)^*^	2.17 (0.85–5.55)
III	1.76 (1.12–2.77)^*^	2.13 (1.14–4.01)^*^	1.48 (0.88–2.50)
IV	2.34 (1.33–4.12)^*^	3.51 (1.72–7.15)^*^	1.72 (0.80–3.70)
V	0.99 (0.14–6.74)	-	1.38 (0.20–9.66)
VI	1.78 (1.16–2.72)^*^	1.89 (0.87–4.10)	1.79 (1.04–3.07)^*^
VII	0.77 (0.58–1.02)	0.86 (0.49–1.52)	0.73 (0.51–1.06)
VIII	1.41 (1.00–1.99)^*^	2.05 (1.30–3.22)^*^	1.03 (0.62–1.72)
Total	1.30 (1.05–1.60)^a^^*^	1.74 (1.30– 2.35)^a^^*^	1.09 (0.86–1.38)^b^

a *Adjusted for age, sex, and group*.

b *Adjusted for age and group*.

* *Groups were significantly different by multivariate logistic regression analysis, P < 0.05*.

*(-):the sample size was too small to display the statistics results*.

# *Group I (large coastal city), Group II (upper class/large city), Group III (middle class/city), Group IV (lower class/city), Group V (upper class/rural), Group VI (middle class/rural), Group VII (lower class/rural), and Group VIII (western/lower class/rural). Group I included the nine largest cities (Beijing, Shanghai, Tianjin, Shijiazhuang, Shenyang, Dalian, Jinan, Qingdao and Nanjing) and Group II, represented the upper urban class. Group VIII constituted the other extreme: rural regions in western provinces, home to the lowest SES class*.

## Discussion

In the present study, by using data from the 2014 CNSSCH covering 26 provinces and four municipalities in China, not only were the differences in the prevalence of anemia among school-aged children by geographic groups, sex, and stunting status documented, but also the major associations between anemia and stunting in school-aged children. On the whole, the prevalence of anemia was significantly higher in girls than in boys, and in addition there was low prevalence of anemia in areas with good economic conditions. However, some regions, such as the poorer rural setting of Group VII and middle-class cities of Group III might be overlooked.

Researchers have pointed out that geographic disparity was related inversely to SES, to wealth at a household level, and to income and education at an individual level (2, 22, 23). Areas with SES were often geographically good, and also had better health care facilities and services (24). Compared to rural areas, individuals living in better SES have a greater availability of food and better housing, electricity, piped water, sanitation, and transportation. Moreover, these populations usually have a higher educational level, economic status, and employment opportunities. Studies in Indonesia have found child stunting to be associated with poor health care practices, inadequate sanitation and water supply, food insecurity, and low caregiver education (25). Sankar Goswmai's study used the wealth index to reflect the state of SES and the results indicated that, relatively, the poorest area had the highest prevalence of anemia (OR = 2.033, 95% CI:1.71–2.22), while the richer area had lowest prevalence of anemia (OR = 1.183, 95% CI:1.14–1.32) (26). In groups with these good economic development indicators, the prevalence of anemia was low; the study mentioned that a child living in a household in the lowest wealth quintile was 21% more likely to be anemic than were those in the highest wealth quintile (27), which is consistent with the present study. In Group I (large coastal city) and Group II (other upper class/large city), the economic development is relatively high and anemia prevalence is relatively lower than other groups. High quality living environments and facilities due to advanced economic development can contribute to anemia reduction among Chinese children (2). A previous study conducted in 32 selected low-income and middle-income countries showed that a child living in a household in the lowest wealth quintile was 21% more likely to be anemic than those in the highest wealth quintile (2). However, the prevalence of anemia did not disappear in Group I and Group II, which indicated that improving the monitoring of anemia, scaling up coverage of prevention, and developing anemia prevention interventions were still needed in China (28, 29).

China has long been concerned about the imbalance of geographic disparity, thus, improving the health of children in rural western China remains a critical health priority (30). The national anemia prevention intervention policy was mainly implemented in the area of Group VIII, i.e., “National Nutrition Improvement Program for Rural Compulsory Education Students (NNIPRCES)” released by the general office of the State Council of China (2011) (31). Even though the prevalence of anemia in Group VIII has been significantly reduced after the implementation of the above policy (32), it is still serious due to its high baseline. Currently, Group VII has replaced Group VIII as the most serious burden of anemia because no extra attention has been given to this group. Other than continuing investment in anemia improvement in rural regions of western provinces, it is recommended that attention should be paid to other areas such as poorer rural settings in China. For example, nutritional education and improvement of diet quality should be included in the content of anemia intervention, to ensure the daily Recommended Dietary Allowance (RDA) of iron-containing foods (meat, milk, etc.) (33). These additional measures may play a more important role in decreasing anemia for vulnerable groups such as children living in western regions, boys with stunting issues, and adolescent girls (14).

Besides Group VII, Group III also had a surprisingly high prevalence of anemia. The higher prevalence of anemia in Group VII indicated that lower economic groups are susceptible and intervention needs to be focused here first, and as such it might be helpful to improve the anemic status by eliminating the geographic disparity. Considering the serious disparity, the government should reconsider the priorities around the anemic burden across the country and develop new strategies and interventions not only to the groups with serious burden, but also those at high risk like Group III.

In the present study, the association between anemia and stunting exists only in boys, but not in girls. Therefore, non-targeted anemia interventions, such as general nutrition improvement, may only work for boys. Ramachandran (34) pointed out that adolescent girls form a crucial segment of the population and constitute the vital “bridge” between the present generation and the next generation. The main reasons for this consequence among girls may be the menstrual bleeding during their puberty and weight loss (35, 36). In order to pursue a slim figure, adolescent girls deliberately lose their body weight by dieting, which may lead to insufficient iron intake (37). Iron deficiency may be a routine consequence of growth and skeletal development (38). Sex differences should be taken into account when implementing anemia interventions. Consequently, it is suggested that all children, especially adolescent girls, need health education due to their vulnerability to anemia (39). Furthermore, reliable measures on the causes of anemia are needed to guide interventions (40).

China, with its significant economic development in recent decades, has experienced epidemiological and demographic transitions which have affected its population's nutritional conditions and produced environments that have contributed to a change in anemia (41). Despite the nutritional transition and improved nutritional status, stunting and anemia remain a major public health problem in China (42). There were some common factors in the occurrence of anemia and stunting. As we all know, the main risk factor for anemia among children is low iron intake at a stage of life in which iron requirement is high. Iron, as an essential trace mineral, is necessary for linear growth and body tissue proliferation (43). Meanwhile, stunting is an indicator of long-term chronic malnutrition, which is primarily caused by insufficient nutrition supply (44). The supply of nutrients includes not only macro-nutrients, but also micronutrients, such as iron (45). One reason why stunting may be related to anemia is that stunting may pick up deficiencies in iron, which are also known to boost the risk of anemia through impaired erythropoiesis and oxidative stress pathways (44). Especially for children in groups with high prevalence of stunting, all age-groups of children were vulnerable groups for anemia (46). In addition, it is worth noting that school-aged children in Group III (middle class/city) in China have been facing the double burden of anemia and stunting. Therefore, “the child- and adolescent- specific dietary guidance” is able to guide children to a reasonable quality diet, which refers to the “recommended daily dietary allowances for children and adolescents” established by the Chinese Nutrition Society (47). WHO recommends that a diet containing adequate amounts of bioavailable iron could prevent and control anemia (48). In addition, community-based platforms for nutrition education and government commitment and focus on equity are also important factors that may lead to the implementation of interventions that prevent and treat child stunting (49–51).

There are limitations in the present study. Firstly, it was a cross-sectional study and it cannot infer causality between anemia and stunting. Secondly, samples of capillary blood from the fingertip of each child were collected after discarding the first drop. Capillary blood can't distinguish the type and cause of anemia. Thirdly, iron deficiency and other nutrient indicators were not assessed directly.

## Conclusions

The present study demonstrated a large geographic disparity in anemia in China. Lower anemia is found in better SES groups, while the higher prevalence is shown in poorer SES groups. The prevalence of anemia in Group VIII was not only due to SES, but also stunting. In addition to focusing on the rural western regions, the government should also pay more attention and provide more resources to the population in middle class cities and lower-class rural areas. The present findings imply that previous measures aimed at improving anemia, regardless of sex and with a limited focus on school-aged children in poor groups, may not be comprehensive enough to tackle the anemia problem in China. Specific strategies and interventions should be developed for children in susceptible groups, and especially for girls.

## Data Availability Statement

The datasets generated for this study are available on request to the corresponding author.

## Ethics Statement

The project was approved by the Medical Research Ethics Committee of Peking University Health Science Center (IRB00001052-18002).

## Author Contributions

J-YW and YS conceptualized and designed the study and completed the statistical analyses. J-YW drafted the initial manuscript and reviewed and revised the manuscript. YS and YM designed the study and collected the data. D-ML and YS assisted with the statistical analyses. YM, JM, PL, BD, P-JH, D-ML, JD, and YS critically reviewed and revised the manuscript. All authors were involved in writing the paper and had final approval of the submitted and published versions.

## Conflict of Interest

The authors declare that the research was conducted in the absence of any commercial or financial relationships that could be construed as a potential conflict of interest.
