# Supplementary material for: Reducing Anemia Among School-Aged Children in China by Eliminating the Geographic Disparity and Ameliorating Stunting: Evidence From a National Survey
Source: Front Pediatr. 2020 May 12;8:193. doi: 10.3389/fped.2020.00193 (PMC7235374; doi:10.3389/fped.2020.00193)
Supplement: Supplementary file 1 [file Table_1.pdf]

Table S1 The prevalence of anemia stratified by geographic group and sex among Chinese school-aged children in 2014

Group#	Total	All		Male		Female	
	N	N	%	N	%	N	%
I	5934	306*	5.2	99*	3.4	207	6.9
II	9133	697*	7.6	303*	6.6	394*	8.6
III	9885	1014*	10.3	382*	7.7	632*	12.8
IV	10654	831*	7.8	367*	6.9	464	8.7
V	5978	359*	6	118	4	241	8.1
VI	8939	715*	8	303*	6.8	412*	9.2
VII	16272	1955*	12	725*	8.9	1230*	15.1
VIII	4320	427*	9.9	183*	8.5	244*	11.3
Total	71115	6304	8.9	2480	7	3824	10.8

# Group I (large coastal city), Group II (upper class/large city), Group III (middle class/city), Group IV (lower class/city), Group V (upper class/rural), Group VI (middle class/rural), Group VII (lower class/rural), and Group VIII (western/lower class/rural). Group I included the nine largest cities (Beijing, Shanghai, Tianjin, Shijiazhuang, Shenyang, Dalian, Jinan, Qingdao and Nanjing) and Group II, represented the upper urban class. Group VIII constituted the other extreme: rural regions in western provinces, home to the lowest SES class.

* Groups were significantly different by multivariate logistic regression analysis, $P<\alpha'$.
