# Supplementary material for: Reducing Anemia Among School-Aged Children in China by Eliminating the Geographic Disparity and Ameliorating Stunting: Evidence From a National Survey
Source: Front Pediatr. 2020 May 12;8:193. doi: 10.3389/fped.2020.00193 (PMC7235374; doi:10.3389/fped.2020.00193)
Supplement: Supplementary file 2 [file Table_2.pdf]

Table S2 The moderate/severe prevalence of anemia stratified by geographic group and sex among Chinese school-aged children in 2014[1]（%）

Group#	Male		Female	
	Mild	Moderate/Severe	Mild	Moderate/Severe
I	4.6	1	5	1.9
II	5.3	2	5.6	3
III	4.1	2.4	8.4	4.4
IV	2.7	2.8	6	2.7
V	4.2	1.2	6.1	2
VI	5.4	2.6	5.9	3.4
VII	4	3.5	10.4	4.7
VIII	4.4	4.5	6.9	4.4
Total	2.4	2.6	7.3	3.5

Group I (large coastal city), Group II (upper class/large city), Group III (middle class/city), Group IV (lower class/city), Group V (upper class/rural), Group VI (middle class/rural), Group VII (lower class/rural), and Group VIII (western/lower class/rural). Group I included the nine largest cities (Beijing, Shanghai, Tianjin, Shijiazhuang, Shenyang, Dalian, Jinan, Qingdao and Nanjing) and Group II, represented the upper urban class. Group VIII constituted the other extreme: rural regions in western provinces, home to the lowest SES class.

[1] Chi-square test was used to assess the difference of the prevalence of anemia among different Groups, the inspection level was adjusted by Bonferroni method [α'=2α/k(k-1), k=7].

* Groups were significantly different by multivariate logistic regression analysis, P<α'.
