# Supplementary material for: Reducing Anemia Among School-Aged Children in China by Eliminating the Geographic Disparity and Ameliorating Stunting: Evidence From a National Survey
Source: Front Pediatr. 2020 May 12;8:193. doi: 10.3389/fped.2020.00193 (PMC7235374; doi:10.3389/fped.2020.00193)
Supplement: Supplementary file 3 [file Table_3.pdf]

Table S3 The prevalence of anemia stratified by geographic group, age and sex among Chinese school-aged children in 2014

Group#	7				9				12				14			
	Boys		Girls		Boys		Girls		Boys		Girls		Boys		Girls	
	N	%	N	%	N	%	N	%	N	%	N	%	N	%	N	%
I	25	3.3	28	3.7	21	3.0	26	3.5	34	4.5	67	8.9	19	2.5	86	11.5
II	77	6.7	79	6.9	86	7.5	64	5.6	91	8.0	107	9.3	49	4.3	144	12.7
III	130	10.5	151	12.1	115	9.3	137	11.3	100	8.1	165	13.3	37	3.0	179	14.6
IV	134	10.1	125	9.4	74	5.6	75	5.6	104	7.9	118	8.9	55	4.1	146	10.9
V	38	5.1	44	5.9	27	3.6	36	4.8	40	5.4	80	10.7	13	1.7	81	10.8
VI	123	10.8	125	11.2	95	8.3	84	7.3	72	6.5	104	9.5	13	1.2	99	9.0
VII	286	14.0	306	15.0	161	7.9	221	10.9	186	9.2	325	16.0	92	4.5	378	18.7
VIII	60	11.1	72	13.2	41	7.6	48	8.9	54	10.0	57	10.8	28	5.2	67	12.3
Total	873	9.8	930	10.4	620	7.0	691	7.8	681	7.7	1023	11.5	306	3.5	1180	13.3

Group I (large coastal city), Group II (upper class/large city), Group III (middle class/city), Group IV (lower class/city), Group V (upper class/rural), Group VI (middle class/rural), Group VII (lower class/rural), and Group VIII (western/lower class/rural). Group I included the nine largest cities (Beijing, Shanghai, Tianjin, Shijiazhuang, Shenyang, Dalian, Jinan, Qingdao and Nanjing) and Group II, represented the upper urban class. Group VIII constituted the other extreme: rural regions in western provinces, home to the lowest SES class.
