# Supplementary material for: Reducing Anemia Among School-Aged Children in China by Eliminating the Geographic Disparity and Ameliorating Stunting: Evidence From a National Survey
Source: Front Pediatr. 2020 May 12;8:193. doi: 10.3389/fped.2020.00193 (PMC7235374; doi:10.3389/fped.2020.00193)
Supplement: Supplementary file 4 [file Table_4.pdf]

Table S4 The prevalence of anemia stratified by stunting status, age and sex among Chinese school-aged children

Age	Boys						Girls					
	Non-stunting		Stunting		χ^2	*P*	Non-stunting		Stunting		χ^2	*P*
	N	%	N	%			N	%	N	%		
7	866	9.8	7	7.4	0..59	0.599	913	10.3	17	20	8.44	0.007
9	612	6.9	8	10.1	1.22	0.263	674	7.7	17	12	3.58	0.079
12	654	7.6	27	12.5	7.26	0.013	990	11.5	33	12.5	0.28	0.624
14	278	3.2	28	11.6	49.56	<0.0001	1154	13.3	26	12.7	0.07	0.917
